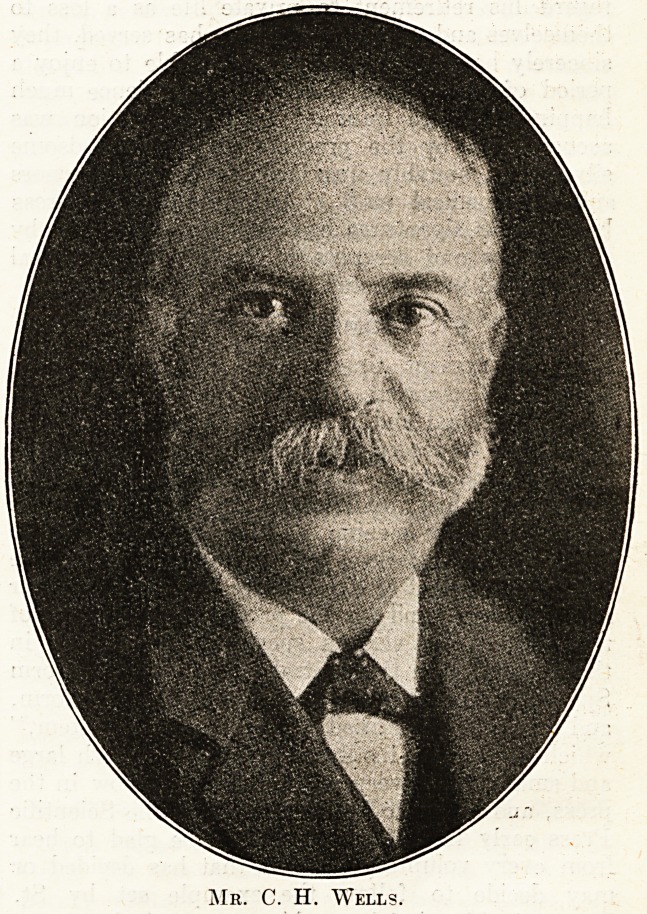# Hospital and Institutional News

**Published:** 1915-12-04

**Authors:** 


### HOSPITAL AND INSTITUTIONAL NEWS.

### GALLANT CONDUCT OF A MEDICAL SUPERINTENDENT.

A sad event occurred on November 21, when four nurses were the victims of an ice accident at Marthall-cum-Warford, a small village near Knutsford, Cheshire. The gallant conduct on this occasion of the medical superintendent of the David Lewis Epileptic Home, Sandlebridge, was referred to at the annual meeting of that institution on the following day, Lord Sheffield, on behalf of the committee, expressing the admiration they all felt at Dr. McDougall's conduct in rescuing the lives of many nurses and patients who had fallen through the broken ice.

### THE COUNTY OF LONDON WAR HOSPITAL

The Mental Hospitals Committee of the London County Council, reporting on the conversion of the Horton Mental Hospital into the County of London War Hospital, state that the buildings have proved very adaptable for their new use and, with the addition of two operating theatres and pack stores, make an admirable general hospital, specially suitable for military patients. The original equipment of the institution required to be supplemented in certain directions to meet the needs of an increased number of cases of physical sickness. The committee correct an impression which has got abroad in some quarters that it is being used as a special hospital for mental and neurasthenic cases. This is not so, the hospital being for the medical and surgical treatment of any sick and wounded soldiers. It has been made a central hospital, and has had affiliated to it ten auxiliary hospitals situated in the neighbourhood, for which it acts as a clearing station, convoys being received there and patients distributed as they arrive at a certain period of treatment or convalescence. For this purpose of reception and distribution a detachment of the Army Service Corps, with a small fleet of motor ambulances, has been attached to the hospital. The general business management of the hospital and the supervision of the staff is in the hands of a sub-committee appointed by the Mental Hospitals Committee of the County Council. It was foreseen that sooner or later the services of men eligible for military duty would be required in other directions, and therefore male assistance was dispensed with as far as possible. The whole of the nursing, with the exception of that in one ward, is undertaken by women.

### A SPLENDID RECORD.

The first convoy of wounded soldiers was received at the hospital on May 20, and since that date 4,175 sick and wounded soldiers have been admitted, of whom 2,240 have been discharged, chiefly to duty or light duty. At the time of the committee's report there were on the books of the hospital 1,915 patients, 1,619 being in residence and 296 in auxiliary hospitals. The committee add that if the small number of deaths (twenty) that have occurred is at all a criterion of the medical, surgical, and nursing activities, they have every reason to believe that the work is being well done. An almoner's department has been established under the supervision of Lady St. Helier, and many ladies, both in the neighbourhood and from London, visit almost daily and distribute comforts not provided by public funds, such as tobacco, etc. Valuable gifts have been made to the hospital, including a wagonette, billiard-tables, and pianolas, while the King and Queen have recently given ten haunches of venison, tobacco, cigarettes, and pipes. The wives and children of soldiers coming from distant parts of the kingdom are provided with comfortable lodgings in the vicinity. Voluntary help has been freely given in the workroom, and some of the residents in the neighbourhood have placed at the disposal of the hospital their motor-cars and carriages.

### LORD DERBY AND RED CROSS MEN.

With characteristic courage and directness Lord Derby has tackled the question of Red Cross workers of military age. He has officially drawn attention to the fact that many men who are eligible for combatant service are employed in various capacities under the British Red Cross and similar agencies, and that they could be and should be replaced by others in the interests of recruiting. We are quite certain that there are a number of men who ought to be serving in the Army but are sheltering themselves behind the Red Cross; whether the number is large we do not presume to say, but Lord Derby is in a position to know this. The French word for this kind of shirker is "embusqué," and there is a very healthy public opinion in France which reprobates such men. It is certainly "up to" the British Red Cross Society and the St. John Ambulance Association to weed their ranks thoroughly, and to secure married men, men over age, and women to fill the places of all who ought to be fighting.

### A HOSPITAL TREASURER DIES OF HIS WOUNDS.

The death has occurred at St. Thomas's Hospital, London, of Lieutenant Spencer Ellwood Barrow, of Lancaster, a member of the Society of Friends, who for three or four years has been the honoured treasurer of the Royal Lancaster Infirmary. Members of his family have been connected with the institution for about a hundred years. His uncle, Mr. Thomas Barrow, was president in 1903, and in appreciation of his work and generous support the committee recently named one of the beds in the institution the "Thomas Barrow" bed. Moreover, one of the deceased's brothers, Dr. W. D. Barrow, is a member of the honorary medical staff of the infirmary. Soon after the outbreak of war, Mr. Barrow, who was in business as an architect, offered himself for enlistment as a private in the Royal Lancaster Regiment—his sense of duty and strong patriotism overcoming his temperament and training as a member of the Society of Friends.

He was, however, offered a commission, which he accepted, and after preliminary training he went to the trenches in France last May. On the very day he got there, and within six hours of being in the trenches, he was severely wounded, his left arm being shattered and the flesh torn and lacerated. He was removed to hospital after some delay, and eventually arrived at St. Thomas's Hospital, where efforts were made to save the arm. He went on well until a few days before his death. He died from the effect of the wounds.

### A QUAKER'S MILITARY FUNERAL.

Military honours were accorded the interment of his remains at Lancaster—the first occasion, writes a correspondent, on which a member of the Society of Friends has been given a military funeral. The president, ex-president, and a number of members of the Lancaster Royal Infirmary committee attended, including the Mayor of Lancaster, the Vicar of Lancaster, and the member for the division, Sir Norval Helme, M.P. Major Bates was in command of the military, and six brother officers of Lieut. Barrow acted as pall-bearers. There was a large number of other officers present, and a strong contingent of military. The firing party was under the command of Lieut. R. J. H. Preston. At the graveside a funeral oration was delivered by Mr. Theodore Neild, a cousin of deceased, and a member of the Society of Friends. The usual three volleys were fired, and the "Last Post" sounded. Among the floral tokens was a beautiful one from brother officers; one from wounded soldiers and nurses at St. Thomas's Hospital; and a third from the president and committee of the Royal Lancaster Infirmary "in remembrance of a devoted colleague."

### SURGEON-GEN. SIR GEORGE MAKINS, K.C.M.G., C.B.

The governors of St. Thomas's Hospital two years ago granted to Sir George Makins an extension for two years of his office of surgeon to the hospital. Sir George was subsequently promoted to be consulting surgeon, and later Surgeon-General, Army Medical Service, and has been abroad in the service of the country since the outbreak of the war. The governors recently invited Sir George to continue on the staff of St. Thomas's Hospital at least until the termination of the war. This unusual step confers deserved honour upon Sir George Makins, of whom the famous American surgeon, Mr. William Mayo, recorded an opinion in one of his books which embodied probably the greatest compliment to an eminent surgeon that has ever been paid by a member of the medical profession. Mr. Mayo declared that to watch Sir George's (then Mr. Makins') work and technique in the operation-theatre made him feel that, should occasion arise, Sir George was the man he would desire to act as surgeon to himself and to members of his family. Our soldiers at the Front are indeed fortunate to have Sir George Makins as their consulting surgeon.

### HOSPITAL SLUGGARDS IN HIGH PLACES.

In the old days it was not difficult to find this type of official. Happily, as the years have passed, their number has materially decreased, but unfortunately the presence of some is still made known by the condition of the affairs of the institution with which they are connected. We had a notable instance of the working of an institution so handicapped brought to us recently. A subscriber, who had worked for and taken a deep interest in the welfare of the particular hospital, gradually increased his subscription to an unusually liberal amount, which he gave regularly year by year. Then a year came when no reminder reached him that his subscription was due and no request for its payment. He, however, paid his subscription, but this action produced no sign of improved business methods, and the institution lost one of its most interested and devoted supporters. He mentioned the fact to us in the hope that we might be able to tell him how to save the institution by securing a reform in its business management. The article entitled "When the Voluntary Hospital Triumphs," on page 203 of the present issue, drives home the lessons of this incident.

### THE LONDON JEWISH HOSPITAL.

Our Note under this head in last week's issue has called forth a remonstrance that the leading Jews are strongly opposed to this hospital, that there are practically no Jewish nurses, and that it cannot be found possible to secure Jewish doctors or surgeons of very high standing on its staff. We inserted the paragraph as an item of news to show the position, though we share the views attributed to the leading Jews and those entitled to speak on the hospital question that there is no justification really to add yet another hospital to those of the Metropolis. It is urged that the London Jewish Hospital can offer to Jews no advantage which they do not already have as patients in existing hospitals, but its patients may be deprived of advantages in the way of medical and surgical treatment which they at present enjoy elsewhere. It is a grave responsibility for anyone to start, much less to force a new hospital upon Londoners, without the most urgent grounds of necessity and administrative completeness. The promoters of the proposed London Jewish Hospital cannot justify their action on either of these essential grounds.

### THE L.C.C. AND MASSAGE ESTABLISHMENTS.

The London County Council has issued, for the benefit of all whom it may concern, a résumé of the powers conferred by Part V. of its General Powers Act (1915), which deals with establishments for massage, manicure, chiropody; light, electric, vapour, and other baths; or similar treatment. After February 1, 1916, no such establishments may be carried on without being registered with the Council (or, in the City, with the Corporation); and the Council has power to refuse such registration, or to cancel it when granted. It is important that all persons affected by this Act should note that after February 1 it will be an offence against the law to publish or display an advertisement of any such institution seven days after publication in the *London Gazette* of the cancellation of a licence. The voluntary hospitals, the rate-supported and other public hospitals, and institutions carried on by registered medical men under certain conditions, are exempt from the provisions of this Act.

### TUBERCULOSIS WORK IN STAFFORDSHIRE.

THAT anti-tuberculosis work is not everywhere at a standstill is seen from the opening of Groundslow Sanatorium, which is now furnished and prepared for patients, according to the arrangements entered into by the Staffordshire, Wolverhampton, and Dudley Joint Committee for Tuberculosis. Some score of patients on the waiting list are thus being provided for, all of whom are women. The joint committee has decided to postpone the preparation of plans for the proposed sanatorium at Lower Penn; while in connection with the Moxley Sanatorium, under the same authority, a medical superintendent has been appointed. In the latter connection it is interesting to add that the gentleman appointed was said by a medical man present at the committee meeting which discussed the appointment to be required for the Army if medically fit for such work. To this suggestion the clerk replied that he understood the new medical superintendent to be ineligible for Army service, since his father was stated to be an Egyptian and his mother a Frenchwoman. The appointment was eventually confirmed. The above questions show that the profession generally, even when concerned to fill vacant posts, bears strongly in mind the medical needs of the Army.

### BOARDS OF GUARDIANS AND THE CRY FOR ECONOMY.

IT hardly seems germane to the duties of Boards of Guardians actively to concern themselves with political controversial matters, but that such activity is not always alien to them is seen in the discussion by the Keighley Board of a resolution of the Lewisham Guardians recommending a reduction in the salaries of Cabinet Ministers. One speaker at Keighley declared that the salaries of British Cabinet Ministers were higher than any others in Europe—a fact, by the way, which testifies to the purity of the British system of government. The motion supporting the resolution of the Lewisham Guardians was carried. Since the effect of such resolutions by such bodies on matters of this kind is negligible, it may seem strange to allude to this matter. But there is a fact to remember which the Lewisham and Keighley Guardians seem to forget. It is this. Boards of Guardians are entrusted with the spending of public money, which, since it involves the maintenance of public services and the public health, it is desirable should be spent, though the only "profit" they show is a clean bill of health or a low death-rate. Such vital services are sometimes foolishly called unproductive expenditure. In these days, therefore, when the cry for retrenchment and economy is indiscriminately raised, public officials like Boards of Guardians should be cautious in lending their voices to it—since the policy of retrenchment is one which may rob them of some of their most useful work. And since the salaries of Cabinet Ministers are not their affair, they had better leave the subject alone for their own sakes.

### A LOSS TO GUY'S HOSPITAL.

BY the retirement of Mr. C. H. Wells, Guy's Hospital loses a servant whose record places him very high indeed among the institutional organisers of the Metropolis. It was in 1880 that Mr. Wells first became connected with Guy's, as clerk to the Dean of the School, in which capacity he rendered

MR. C. H. WELLS.

invaluable services to three successive Deans. These services are duly recapitulated in the last issue of the *Guy's Hospital Gazette*; but even more important were his activities after he was appointed secretary to the treasurer of the hospital itself, and subsequently as clerk to the hospital. During the long period that he has filled these offices Mr. Wells has been a driving force in the financial regeneration of the hospital. He was the treasurer's trusted helper when the Sustentation Fund, the Re-endowment Fund, and others were initiated—funds which, in combination, are estimated to have brought in nearly a million to the hospital's exchequer. These services have been suitably acknowledged by all connected with Guy's, and Mr. Wells carries also with him in his retirement the good wishes of institutional men from wider circles than those in which his work has lain.

### OFFICIAL RECOGNITION.

Thus at a general court of governors held on November 5, it was resolved: "That the best thanks of the governors are due to Mr. C. H. Wells for the able and efficient manner in which he has uniformly performed his duties as secretary to the treasurer, and latterly as clerk to the hospital, together covering a period of twenty years; and, further, that he carries with him, on leaving the positions which he has so satisfactorily filled, the esteem and good wishes of all who had occasion to transact official business with him. While they regard his retirement to private life as a loss to themselves and to the hospital he has served, they sincerely hope that he will now be able to enjoy a period of well-earned rest, and experience much happiness in the future." This resolution was accompanied by the presentation of a handsome silver tray, suitably inscribed, from the governors and the medical staff. An illuminated address bearing 118 signatures has also been presented by the officers and servants of the hospital, medical school, and nursing staff.

### ST. THOMAS'S HOSPITAL.

At the present time, when economy is being preached throughout the land as the one policy every individual and institution must pursue, it is interesting to record that Sir William Plender, the eminent accountant, who stands at the head of his profession, has undertaken to investigate the method of book-keeping at this hospital, and to make recommendations for the improvement of the system of control of expenditure by the treasurer and almoners, and for the proper co-ordination of the work of the various spending departments in the hospital with the central office. The Uniform System of Accounts has now assumed a final form, and a new book entitled "The Uniform System," which deals with institutional accounts, both large and small, from every point of view, is now in the press, and is due to be published by The Scientific Press early in 1916. We should be glad to hear from every voluntary hospital that has decided or may decide to follow the example set by St. Thomas's Hospital by making a practical attempt to improve its system of control of expenditure and the co-ordination of the work of its spending departments with the central office.

### A "THREATENED INJUSTICE" AT BIRMINGHAM.

The Infirmaries Committee of the Birmingham Board of Guardians have been considering what economies it is possible to effect in connection with the expenditure at Selly Oak Infirmary. The dietary, so far as meat and tea are concerned, is reported to be curtailed; gas fires are taking the place of stoves, and the lighting system is being reorganised. The question of Christmas allowances also came up for consideration, but after suggestions had been made for curtailing the customary presents of tobacco and tea to the inmates of the institution, it was ultimately decided to make no difference from the allowances accorded last year. On the other hand, the need for economy did not influence the Guardians to accept a suggestion emanating from the Army Council, which proposed that the Poor-Law probationers (who were accepted for service when the Dudley Road institution was handed over to the military) should continue to be paid at the previous rates. They receive the same salary—namely, £20 per annum—as the V.A.D. probationers, but it was proposed that they should wear their old uniform and receive an allowance of £3 a year for it, as compared with the allowance of £4 made to the V.A.D. probationers. The Board, on the recommendation of the Infirmaries Committee very properly decided to make a strong protest against giving V.A.D. probationers better conditions of service than those offered to Poor-Law probationers, all of whom had had previous training. The Committee expressed themselves strongly upon this "threatened injustice" to their nurses, and it is to be hoped that their protest will lead to the abandonment of the proposal.

### LYING-IN HOMES: THEIR INSPECTION AND SUPERVISION.

It is most important that the inspection and supervision of lying-in homes and massage establishments should be absolutely impartial and efficient. In order to guarantee this it seems to us essential that both shall be undertaken by the London County Council, and that that body shall not avail itself of the power to delegate any portion of this responsible work to Borough Councils. Experience has led those who have the most intimate and practical knowledge of the working of the Metropolitan Borough Councils to feel that if efficiency in regard to inspection, supervision, and kindred work is to be maintained at the highest point, the interests of the citizens demand that the Borough Councils shall have nothing whatever to do with it. We venture to voice this feeling as a protest to the *ex parte* statements circulated by the Standing Joint Committee which represents the London Borough Councils. Some of those statements which seek to claim that the Committee of the House of Lords had in mind that the Borough Councils and their officers are the proper authority to inspect and supervise lying-in homes are not justified, in our view, by the proceedings which took place in Committee when the Bill was under consideration. In any case the interests of the citizens throughout the Metropolis require that this work shall be undertaken and continued uninterruptedly by the London County Council and its officers.

### THE VALUE OF REGIMENTAL CHIROPODY.

For many months past members of the Incorporated Society of Chiropodists have been giving gratuitous treatment to soldiers at the clinic of the society, 1 Silver Street, Bloomsbury, which is open for this purpose every evening with the exception of Monday, and large numbers have attended for the treatment of various foot conditions coming within the scope of the chiropodist. The attention of the War Office was drawn to the usefulness of this special work—for there is no doubt that careful attention to these ailments saves much suffering and discomfort, with consequent muscle strain in marching—and arrangements were made with the president of the society whereby he and other members were engaged to attend at some of the hospitals in the Eastern Command—Woolwich, Chatham, Dover, and Harwich. In addition to treating the patients, they organised classes and gave instruction to selected men to enable them to act as battalion chiropodists.

### A RECRUITING PROBLEM.

A doctor who signs himself a "Recruiting Medical Officer" writes to the *Times* suggesting a relaxation of the standard of vision at present demanded for recruits; the particular reform he suggests is that any man whose vision can be brought to "half normal" by the aid of glasses should be regarded as fit—as far as his eyesight is concerned. By the expression "half normal" he presumably means 6/12, a standard of vision which is, in fact, nearer 95 than 50 per cent. of the normal acuity. We were under the impression that for some years recruits had been allowed the aid of glasses in passing the doctor. Certainly this was the case in the Territorial Force, and it would indeed be strange if a different standard prevailed among those comrades with whom they fight side by side. If, however, it is the fact that glasses are not allowed for the New Armies, we favour the suggestion of the Recruiting Medical Officer. The one drawback is that any man wearing glasses who gets "fed up" for any reason has only to break or lose them in order to get sent down to the base; but the proportion of men who would do this is probably small enough to be negligible.

### AFTER-CARE ORGANISATION IN BRISTOL.

In the report of the Medical Officer of Health for Bristol an interesting account is given of the organisation of the after-care of consumptives. The Bristol Civic League, which controls the work, possesses five district committees, to which patients are affiliated according to their place of residence. In addition to such attention as may be given by the tuberculosis nurses and the city health visitors, a worker is appointed by the committee to visit the patient's home. When necessary, not only clothes and fares for sanatoria, but beds and bedding, are given, and insurance arrears are paid. The League receives applications for assistance from the clergy and others, and cases are referred to it by the Health Committee and similar bodies. The District Committee further sends a full report on each patient to the Tuberculosis Committee of the League, which is a body consisting of the chief tuberculosis officer, the medical officer of health, and the Clerk to the Guardians. Grants both of money and clothes are made by the Tuberculosis Committee, whose chief value is in the link which it affords between the voluntary and public agencies of the city. In spite of this organisation, complaint is made that some patients may never get referred to the Civic League. Such a defect, however, could easily be got over by judicious publicity, and any District Committee should be made responsible for seeing that it is possessed of knowledge of every case requiring after-care in its own area. With the organisation already in train this should not prove a difficult matter.

### WHY THE DISPENSARY IS NOT SUPERSEDED.

The life of the dispensary seemed to be threatened more than that of any other institution by the advent of the Insurance Act, and the fact that the dispensary has survived, and therefore proved the prophets apparently to have been in the wrong, really shows that the Act has not done what it professed to do. The patient in the ordinary minor ills of life, for which the dispensary made provision, was supposed to be provided for by the panel system. He paid his tax and nominally had his own doctor always ready to attend him. Yet we find the dispensary still in existence, and actually claiming support on the ground that it is the only institution which visits patients in their own homes. If this does not mean that the panel doctor's visits are insufficient or do not provide what the patient requires it means nothing. The existence of the dispensary is the measure of the ineffectiveness of the panel system. For an example we need go no further than Leeds, where the Public Dispensary, by developing its special departments, has supplemented the work of the Insurance Act. The work of the dispensary in the past year is remarkable in its scope and organisation. Besides a system of home-visiting—now curtailed, it is true, by a shortage of staff—soldiers and recruits have been treated in the dental department, and minor accidents of various kinds have been dealt with; the Belgian refugees have been supplied with medicines free of charge; rooms in the dispensary have been placed at the disposal of the general infirmary, whose out-patient department has been converted into wards for soldiers.

### MILITARY HOSPITALS ON THE SOUTH COAST.

It is stated that proposals are made by the War Office for the establishment of some eight temporary military hospitals on the South Coast, and in that connection we learn that suitable sites have already been inspected at Worthing, Littlehampton, and Bognor. It is thought that provision for some 30,000 wounded will be made, each hospital receiving about 4,000 men, and that building operations and arrangements will be so pushed forward that the hospitals will be ready for use at the beginning of the New Year.

### SHEFFIELD STUDENTS AND LATIN.

The decision of Sheffield University no longer to insist on Latin as an indispensable part of the preliminary education of aspirants for medical degrees there may or may not be a step to meet the predicted shortage of doctors after the war. It is a proposal which is certain to arouse both hearty commendation and violent protest in the ranks of the already qualified and amongst educationists at large. For our own part we see no great harm in abandoning a requirement which has, we suspect, been honoured much more in the letter than in the spirit at this University, provided that steps are taken to replace it by an increased standard of proficiency in other branches of education, especially in a knowledge of our mother tongue. If a medical student has time and opportunity before commencing his professional studies to learn properly only one language, we prefer that that language should be English, but Latin has great value for the intelligent student.

### SUNDERLAND ROYAL INFIRMARY.

How far the "temporary" additions to our hospitals necessitated by the presence of soldiers will prove permanent extensions of accommodation is a question raised by the new pavilions opened at Sunderland Royal Infirmary. A second request from the military authorities that the infirmary should receive further batches of wounded has led to the erection of four new pavilions where open-air treatment can be given. Each pavilion has been presented by an individual donor, and has been built from the designs of Dr. William Robinson, senior surgeon to the institution; the general plan is that which Dr. Robinson has employed at Stanhope Sanatorium. The four pavilions, which have cost £600 to build and equip, accommodate six patients apiece, and are situated in pairs at the extremities of the two wings of the infirmary, a plan by means of which the sanitary blocks attached to these wings can be conveniently made use of by the patients in the new pavilions. Mr. Ezra Miller, a member of the house committee, has supervised the work, and the chief points of the construction are as follows: Each of the pavilions, which, as already stated, are built in pairs, measures sixty feet in length and is fifteen feet broad. Set on a concrete floor, raised about two feet above the ground, and enclosed at the ends with glass, the roofs project over the semi-open fronts, over which protecting curtains can be drawn in severe weather. This addition to the Royal Infirmary is sufficiently substantial to tempt the authorities to regard it as a permanent extension. From that point of view it will be seen that the cost of building and furnishing the new pavilions works out at £100 per bed.

### THE LIMITATION OF HOSPITALS IN LEEDS.

That the new scheme for the opening of further war hospitals is beginning to recognise the necessity for co-ordination as well as extension may be judged from a letter received by the Holbeck Board of Guardians from the Army Council. The object of the letter was to state that Holbeck Workhouse would not be needed as a military hospital, but it went on to say that it had been found necessary to limit the number of hospitals in Leeds, having regard to the large schemes now in process of formation. As regards Poor-Law buildings, it seems that staff difficulties stand in the way of any more being taken over as military hospitals, and it is well for Guardians, in view of the sacrifices which many Boards have been called on to make, to note that there is a limit which the Army Council recognises to the extent to which their accommodation in Poor-Law institutions will be made use of.

### INDISCIPLINE IN SANATORIA.

We may follow up our recent account of the "revolt" of sixteen patients from Moxley Sanatorium, by recording the verdict on the situation arrived at, after consideration and report, by the Staffordshire, Wolverhampton, and Dudley Joint Committee for Tuberculosis. The house committee of the sanatorium, having received the medical officers' report, interviewed the patients, and after considering their complaints, unanimously expressed the opinion that the medical officer "had acted solely in the interests of the patients, and the proper government of the institution." The report was passed. Since a few of the patients apologised and sought readmission, it is clear that the "revolt" was the work of two or three ringleaders, who were probably suffering from the *ennui* that often attends the semi-convalescent life of the more or less active consumptive. Such men will often make a change in the administrative staff the excuse for attempting a breach of discipline; and the ever-present possibility of the presence of such persons among the patients is one of the difficulties with which the sanatorium medical officer, particularly in a new post, has to contend. To vindicate his authority with firmness and also tact is not always so easy as it sounds, and the medical officer at Moxley Sanatorium is to be congratulated on the report which his action has gained for him.

### THIS WEEK'S DRUG MARKET.

There are no signs of any cessation in the steady advance in the prices of the commonly prescribed synthetic drugs, and week by week higher quotations are registered for several of the medicinal products falling in this category. Thus barbitone, salicylic acid, and phenacetin are again dearer. Acetyl-salicylic acid also has an upward tendency in price, which is rather curious in view of the fact that this drug is now in much more plentiful supply. Some of these drugs are only procurable in very small quantities, and it will only be by the exercise of the greatest economy that a famine in these useful products will be averted. A large business has been done in camphor at dearer rates, but it is doubtful whether values will advance much further. The decline in the price of quinine has been more pronounced, and it would probably be advisable to delay making purchases for the present. Ipecacuanha is again dearer, in consequence of the increasing requirements of the Army Medical Service for emetine for the treatment of dysentery. The value of menthol is not quite so firmly maintained. Further business has been done in cod-liver oil, buyers having apparently come to the conclusion that it is useless to wait for prices to decline.

## Figures and Tables

**Figure f1:**